# Blood Poisoning

**Published:** 1858-01

**Authors:** J. T. Jameson

**Affiliations:** South Otselic, Chenango County, N. Y.


ART. II.—*Blood Poisoning. Numerous Abscesses.* By J. T. Jameson, M. D., South Otselic, Chenango County, N. Y.

Oct. 31st, 1856, I visited, in consultation with Dr. Bowen, of South Otselic, Miss Beasly, æt. about 15, and received from him the following brief history of the case up to the time of my visit, the doctor having unfortunately taken no notes. The subsequent record of the progress of the case is from my own note book, penned after each visit. The patient has not menstrated, but has always enjoyed good health up to the period of the present sickness.

Dr. B. saw Miss B. for the first time, about the 8th of October, and found her suffering from a general febrile state, the pulse being about 100; tongue slightly coated, and considerable pain in the head; beneath the chin was observed a hard, somewhat painful swelling, but devoid of redness, and which terminated by resolution in about a week; the throat was somewhat sore, attended with difficulty of deglutition, but the patient could not open the mouth sufficiently to permit of any satisfactory inspection of the fauces; there was also bronchitis of a subacute character; one of the toes (the fourth of the right foot) was inflamed and in a state of suppuration, and had been so for several days, the patient not being conscious of having injured it, although, as she had gone barefooted somewhat, it is very likely that such was the case; little attention, however, was paid to the toe, and it was not then regarded as having any connection with the patient's sickness. Complains of some pain at the top of the right shoulder; sleep disturbed, the patient muttering and talking considerably during the same; bowels easily moved, and rather disposed to diarrhœa.

Such were the early symptoms of this case, and they were considered as having their origin most probably in cold, to which the patient had improperly exposed herself.

Under the usual antiphlogistic treatment, with the exception of bleeding, which the state of the pulse did not seem to justify, the febrile state and bronchial affection subsided in about ten days.

About a week after Dr. B.'s first visit, a rash, bearing much resemblance to that of scarlatina, appeared over the whole surface of the body, having small reddish looking pimples interspersed: this rash continued in a florid state for two or three days, and then gradually disappeared, unattended with desquamation.

As already stated, the patient, about the time of Dr. B.'s first visit, complained of pain at the top of the right shoulder, but no examination of the part was made until about four days afterwards, when, on inspection, it was found to be slightly swollen, and soon presented the appearance of an ordinary abscess, and being opened, discharged healthy looking pus, the incision speedily closing, and no further collection of pus occurring. Simultaneously with this, a second abscess, about the size of a walnut, was observed a little above the left mastoid process, not manifesting redness, or pointing, fluctuation being however, evident, and remaining so to the present date, the abscess being, Dr. B. considers, somewhat diminished in size.

About the time of the healing of the abscess on the shoulder, a third appeared on the left forearm in the interosseous space, not attended with much pain, and being opened, discharged healthy looking pus, the wound closing in about four days, but subsequently opening again, and after discharging a little, it closed permanently.

On the 24th of October, the patient having complained of some pain in the back, a fourth abscess was found, on examination, to have formed on the left side of the spine and near the inferior angle of the scapula, without redness of skin or pointing, the matter being manifestly at a considerable depth. On this day, also, three additional abscesses were discovered to be forming, namely, between the right axilla and mamma, on the right forearm in the interosseous space, and on the left side of the neck posteriorly. There was no redness of the skin or pointing connected with these abscesses.

25th. An incision was made into the abscess on the left side of the spine, healthy looking pus being discharged, and the wound quickly closing.

26th. Fluctuation being manifest near the incision of yesterday, another was made, pus of like appearance being discharged, and the wound closing as before.

27th. Opened the abscess between the right axilla and mamma.

29th. Incision of yesterday closed, and fluctuation being apparent near it, another was made, and considerable pus evacuated.

Those on the forearm and left side of the neck have not yet been opened.

During the formation and continuance of the above abscesses, slight febrile action has existed a principal part of the time, the pulse ranging from 110 to 125, and mostly rather weak and small; copious sweats, especially during sleep; progressive emaciation and debility; tongue thickly coated, until the 25th of October, when it began to clean and put on a red and glazed aspect; bowels have manifested a disposition to diarrhœa, which has, however, been readily controlled by anodynes; the state of the lungs has been gradually improving.

On the 25th of October, the patient began to take quinine, strong beer and brandy, with the effect of checking the sweats in a marked manner, and under their use, with occasional anodynes, a little infusion of quassia, and nutritious diet, there has been a gradual improvement as respects the constitutional symptoms. The above completes the entire history of the case so far as I could ascertain it from Dr. B.

*Present State.* The countenance of the patient indicates prostration, and the emaciation is considerable; pulse 110, and small; tongue almost clean, rather too red, and somewhat glazed; bowels disposed to diarrhœa, but easily controlled by opiates; not much appetite; coughs and expectorates a little; breathing about normal, and the thorax sounds well on percussion. beneath the clavicles; slight flushes of fever during the day, but the sweating has almost ceased; on the left side of the spine, and a little below the inferior angle of the scapula, there exists a swelling about the size of the hand, in which fluctuation is evident, but without redness of the skin or pointing; there are the marks of two incisions made by Dr. B. A lancet was introduced, and, perhaps, a third of a teacupful of healthy looking pus evacuated. A little below the right axilla, is a second collection of matter similar to the first, only smaller and fluctuation less decided, which presents the marks of two former incisions; a third incision was made, but failed to reach the pus. On the right forearm, in the interosseous space, is a third swelling, diffused, rather hard, somewhat tender on pressure, skin not red, and fluctuation very obscure. On the left side of the neck posteriorly, is a fourth swelling similar in character to the last mentioned one. On the left forearm, in the interosseous space, is the cicatrix of the abscess opened by Dr. B. at an early period of the disease, and which appears to be now quite sound.

Bread and milk poultices are being applied to the abscesses opened, and a stimulating liniment to the others. She is taking about two grains of quinine every four hours in strong beer, brandy occasionally, diet nutritious, and morphine according to circumstances. I recommended a continuance of the same, with the addition of two grains of hydriodate of potash three times a day.

Nov. 2d. General aspect of the patient rather improved; tongue clean, not so red, and less glazed; pulse 110, rather fuller and stronger; slept well last night.

The patient yesterday directed the attention of Dr. B. to the left gluteal region where she experienced pain, and on inspection there was found a swelling over the gluteus maximus similar in character to the others; no particular change in the other swellings; the third incision made into that on the back has closed, and pus again collected; the abscess on the scalp is being gradually absorbed.

Cont. Med.

5th. Patient has been about the same since last visit. Pulse 120, and rather small; tongue clean; coughs and expectorates a little; fluctuation being very distinct over the left gluteus, Dr. B. yesterday made an inciand evacuated about three ounces of healthy looking pus; fluctuation is very distinct in the right forearm not far from the wrist, and on being opened, the abscess was found to extend upward near to the elbow, the pus being evidently deep seated in the intermuscular cellular tissue, and having gradually worked its way downward; that below the right axilla, was opened for the third time, a tent being introduced with a view to keep the wound open, and I may as well say once for all, that from this time tents were regularly introduced into the different abscesses, being found to answer admirably in maintaining an open state of the wound until the abscess became soundly healed.

Cont. Med.

9th. Since last visit three fresh abscesses have formed, one on the left leg in the interosseous space, the second on the inner side of the left thigh near its junction with the pelvis, and the third on the right side of the neck. Dr. B. opened the second one yesterday, and the first was opened to-day, a considerable quantity of bloody looking pus being discharged. The other abscesses opened have mostly healed, with the exception of that on the right forearm, where a sinus extends a good portion of its length; a compress and bandage were applied with a view to its obliteration. The debility and emaciation have increased; pulse has varied from 110 to 120; the tongue has become covered on the posterior half with an apthous like coat; no appetite; bowels about regular; the abscess beneath the scalp is almost absorbed; there has been a little more cough and expectoration during the last three or four days. With the formation of fresh abscesses, and the drain from those already opened, it would seem that the patient must soon sink.

Omit pot. hydriod., and give quinine every two hours in strong beer; continue brandy, and give in addition ℥ss of wine every four hours.

11th. Patient appears somewhat improved, the pulse being stronger, and strength increased, as manifested by her disposition to help herself in turning in bed, and ability to do so; pulse about 120; hectic flush on left cheek; still coughs and expectorates, the cough being quite loose and expectoration easy; tongue clean; opened the abscesses on the back and right side of the neck, warm water dressing with oiled silk, being subsequently applied; the remaining abscesses are doing well, being all about healed, with the exception of that on the right forearm, and the compress and bandage seeming to be acting favorably upon it, only a few drops of serous fluid being discharged by pressure in the centre of the sinus; no new abscesses, so far as can be ascertained. The wine and more frequent doses of quinine seem to have acted favorably.

Continue the same.

14th. Patient's general condition about the same, but four or five new abscesses are forming, one near the right popliteal space, a second in front of the left elbow-joint, a third in the right lumbar region, and a fourth in the left lumbar region; abscesses opened are doing well.

Cont. quinine, wine, &c.

17th. Opened abscess near the right popliteal space; emaciation and debility seem increasing: the fourth toe of the right foot, which is described in the early part of the case, as being in a state of inflammation and suppuration towards its extremity, has been, for some time past, in a dry scabby condition, and of a darker color than natural; it is now discharging again, and, pressure being made, pus oozes out directly from its extremity, the whole toe, as far as the metatarsal articulation, presenting a dark brown color; the outer aspect of the dorsum of the foot is swollen, but the skin is not red, and no red lines can be discovered on the foot or leg.

Apply a lotion of hydriodate of potash to the toe. Cont. med. and give in addition citrate of iron ter in die.

21st. No particular change since last visit, the patient's countenance and pulse being rather improved; an incision was made into the abscess in the left lumbar reg on, but failed to reach pus, although the fluctuation was very decided.

Apply flax-seed poultice.

On examining the diseased toe more minutely, there was found a mass of fungus granulations projecting from the extremity of it, and observing a gray spot, like a slough, at the centre of the above mass, I touched it, and found that it was a small shell of bone imbedded in the granulations, and removed it; the toe looks better, although retaining the same dark brown color.

Apply arg. nit. to the granulations.

The patient's appetite is improved, and the tongue clean.

Cont. iron, quinine, wine, &c.

23d. General condition about the same. Opened the abscess on the right side, which appeared to be deep among the abdominal muscles, and evacuated a considerable quantity of a rather thinnish pus; another deep incision was made into the one on the left side, but no collection of matter was reached, although the lancet was covered with pus; toe improved, being less dark in color, and the fungus granulations diminished.

Applied arg. nit. again. Cont. med.

27th. No particular change. Abscess on the right side discharges a serous fluid in small quantity on pressure, and that on the left has discharged some pus into the poultices; that near the popliteal space, Dr. B. opened again yesterday, the first incision having closed, the pus, which is of a bloody character, appearing to extend deep beneath the heads of the gastrocnemii muscles. Pulse 120, and small; coughs occasionally, but no expectoration; sweats during sleep; bowels rather disposed to constipation; toe almost cicatrized.

Cont. med.

Dec. 1st. Patient's general condition has rather improved; countenance looking better, and tongue clean and more natural. Opened another abscess this morning a little below the right axilla, and another, but more deeply seated, seems formed in the right lumbar region.

Cont. Med.

9th. Some improvement; tongue clean and natural; appetite pretty good; pulse 120, and Dr. B. states that it has fluctuated from 115 to 130; has a little dry cough; bowels moved once in forty-eight hours; Dr. B. has opened the abscess in the right lumbar region; that near the right popliteal space has ceased to discharge, and appears to be soundly healed; that in front of the left elbow advances very slowly, the matter being underneath the tendon of the biceps muscle, and giving an obscure feeling of fluctuation; the elbow is considerably flexed, and cannot be extended.

Cont. quinine, iron, wine and brandy.

14th. Patient seems more prostrated; pulse 130, small and weak; countenance pale and inclining to sallow; tongue clean, and appetite good; has sat up about an hour and a-half; has coughed more, and expectorated some; no fresh abscesses forming so far as known, and those opened have dried up; the swelling in front of the left elbow remains about the same, and although fluctuation was very indistinct, it was deemed best to make an incision, but we did not reach pus; the elbow continues firmly flexed. It ought to have been stated that the right knee continues in a flexed state, in consequence of the abscess in the popliteal space.

Cont. med., and give wine more freely.

26th. Patient has continued about the same since last date, the pulse varying from 115 to 130; coughs occasionally, but no expectoration; tongue clean; appetite pretty good; pulse 120; countenance looks better than at last visit; there has been some discharge from the left elbow, but a swelling still remains there, and the joint is more flexed than it was, and cannot be extended, the wound being still open and discharging slightly. Is using a lotion of hydriodate of potash. Since last visit, an abscess has formed in the interosseous space of the right leg, opened by Dr. B., and seems doing well. Has not been able to sit up the last week, in consequence of the right leg.

Cont. med.

Jan. 1st, 1857. No material change since last visit. Dr. B. states that another abscess formed in the interosseous space of the right leg near the ankle which he opened, and which is now dried up, a second in the right lumbar region which discharged itself by a former incision; there still remains a tumor in front of the left elbow joint, but little painful, and without manifest fluctuation, the joint being flexed and incapable of extension, and the incision almost closed; countenance improved; tongue clean, and appetite pretty good; has considerable dry cough at intervals, and her mother thinks that it has increased the last few days; pulse 115; has continued the use of quinine, beer, wine, iron and brandy.

Cont. ibid.

Jan. 24th. Saw Dr. Bowen, who informed me that the patient at his last visit, three days previously, appeared to be doing well; cough diminished; has gained some flesh, and has sat up as much as two hours; no fresh abscesses; the swelling still remains in front of the left elbow, which is rather more flexed; has been using an ointment of hydriodate of potash to the swelling, but apparently without benefit; try tincture of iodine, and if that fails to do good, a small blister; has been taking a syrup of tamarack bark which has seemingly proved serviceable to the lungs.

Feb. 13th. Dr. B. informed me that the patient has been gradually improving since last date, being now about the house, and able to sit up most of the day; the ointment of hydriodate of potash did not appear to exert any decided influence upon the swelling in front of the left elbow, and the tincture of iodine was then applied with apparent good effect, the tumor gradually subsiding, and she can now extend the elbow nearly to the full extent; the right knee remains somewhat stiff, but is gradually improving; cough gradually disappearing.

March 11th. Dr. B. informed me that our patient was doing remarkably well, having alm st acquired her usual flesh, had lost her cough, and almost regained the perfect use of her left elbow, and right lower extremity. It is only necessary to add, that her recovery was complete and permanent.

*Remarks.* 1. What was the nature of Miss B.'s disease? I do not desire to be dogmatic in supposing that I have rightly designated the complaint, being rather content with having furnished a faithful record of the case, and allowing each of my professional brethren to judge for themselves. It is my opinion, however, that that insignificant toe was the focus et origo mali. On referring to the history of the case, we find that the patient had gone barefooted, and might, consequently, have very readily bruised the extremity of that toe, and yet have thought little of it at the time, and the incident have passed from the memory. However this may be, it is certain that the toe was in a state of inflammation and suppuration prior to the system becoming generally deranged, and several days prior to Dr. Bowen's first visit. It seems further evident from the subsequent history and appearance of the toe, together with the exfoliation of the scale of bone, that the inflammation and suppuration were deep seated, affecting the periosteum, and of an unhealthy character.

My idea, then, of the origin of the disease is, that the patient bruised or otherwise injured the extremity of the toe, which was followed by inflammation extending to the periosteum, or primarily affected the said membrane —that the formation of unhealthy pus ensued—that this pus, not finding a speedy outlet, was taken up into the circulation and poisoned the vital fluid, from whence originated the fever, the cutaneous eruption, the lung affection, and the numerous abscesses, implicating almost every part of the body. The fact of this rash and the soreness of the throat may lead some to think of scatlatina, but several circumstances preclude the entertaining of such an idea: 1. There was no scarlatina in the neighborhood. 2. There were several children in the family, and all remained well during the patient's sickness. 3. The eruption did not appear until the patient had been sick a week or ten days, and the first abscesses had begun to form. 4. The swelling was under the chin, and not at the angles of the jaw. 5. There was no cutaneous exfoliation.

It may be proper to state that Dr. Avery, of South Otselic, who visited the patient once or twice, was disposed to consider the disease as acute periostitis. I could not accord with him in this opinion. I have seen several cases of that disease, and although the periosteum of two or three bones may simultaneously or in succession, become the subject of inflammation, I never witnessed anything like the number of abscesses occurring in the present case.

2d. Some of the abscesses in Miss B.'s case occurred in situations where apparently no bone could be implicated.

3d. In all the cases of periostitis which I have observed, so far as I remember, every successive inflammation of the periosteum of the different bones has ended in death of said bones to a greater or less extent, and exfoliation of the same; whereas in Miss B.'s case, although there was undoubt edly inflammation of the periosteum and exfoliation of bone from the toe, yet of all the subsequent abscesses, several of them deep-seated and assuredly in close approximation to the bones, not the slightest exfoliation of bone occurred from any of them, and they healed in a manner hardly credible under the supposition of the periosteum and bone being affected.

For these reasons, I am compelled to differ from my friend, Dr. Avery, and to adhere to the view above expressed, until further lignt dawns upon my mental horizon.

I think it will be instructive to compare the present case with the interesting one of morbus coxarius, &c., rec rded by Dr. O. C. Gibbs in the September number of the Journal. I am disposed to believe that purulent absorption and blood poisoning occurred in that case. Those who feel interested in the subject will find a very valuable article in reference thereto, in No. 41, November, 1837, of the American Journal of the Medical Sciences, by Dr. Watson, of New York. In this paper, Dr. W. states, "that the doctrine which ascribes the secondary disorders to a vitiated condition of the blood induced by the purulent matter, *or other morbid exhalations of inflamed veins mixing with the blood*, the contaminated fluid thus exciting local inflammation in the parts secondarily diseased, is the doctrine most conformable to facts." And in another place, "for it does not follow because capillary phlebitis leads to the local secretion of pus, that the existence of pus in any part is a proof of previous inflammation there. In the majority of instances, the secondory abscess is, beyond a doubt, connected with local inflammation; but where we see superficial collections appearing suddenly without pain, heat or redness: in a word, without any other of the common signs of inflammation than mere tumefaction, we have good reason to believe that such matter has been deposited independently of inflammatory action; in short that it is an exhalation from the contaminated blood, thrown into the cellular tissue in the same way that serum is thrown into the cells of this tissue in a healthy condition of the system; and that it may be taken as evidence of an effort on the part of the general economy, to unburthen the circulating fluid of a foreign substance, by depositing this substance in places in which it is not so likely to be injurious."

*Treatment.* 2. I am not aware of any specific remedy for this disease; as regards the present case, supposing it to have been one of the kind, a moderate antiphlogistic treatment seemed all that was demanded in the first instance, and all that the constitution would bear; and it soon became manifest that tonics were demanded, and that to a large extent, as the disease progressed. The patient drank many gallons of wine, brandy, and strong beer, besides the quinine and iron, and notwithstanding a liberal administration of each of these articles, and the careful nursing of a good mother, it appeared, once or twice, as though the patient must inevitably sink under the exhausting disease. There seemed to be no other indication, but to sustain the patient, in every possible way, until such times as nature should accomplish the depuration of the vital fluid; and if the case furnishes no other instruction, it cannot fail to impress upon the mind, the importance of never despairing, even in the worst of cases, but of persevering courageously to the end. In relation to the local treatment, the patient might have been saved considerable pain, and we much annoyance, by the earlier adoption of tents. Several of the abscesses appeared not to be circumscribed by adhesive inflammation, and the pus diffused itself in the subcutaneous and intermuscular cellular tissue; the skin was unaffected, and consequently, although fluctuation might be very distinct, the incisions, having to be made through the healthy skin, speedily closed, leading to a re-collection of pus, and further incisions, both of which were almost entirely dispensed with by a careful introduction of tents, and the patient delivered from much additional suffering.

